# The Rolling Stones: A case report of two surgical abdomens linked by migrating gallstones

**DOI:** 10.1016/j.ijscr.2021.105658

**Published:** 2021-02-18

**Authors:** Gerard Lambe, Mark Murphy, Hazel O’Neill, Simon Doran, Noel E. Donlon, Niall McEniff

**Affiliations:** a Radiology Department, St. James’s Hospital, James’s Street, Dublin 8, Ireland; b Radiology Department, The Mater Misericordiae University Hospital, Eccles Street, Dublin 7, Ireland; c Department of Surgery, St. James’s Hospital, James’s Street, Dublin 8, Ireland

**Keywords:** Acute abdomen, Appendicitis, Cholecystitis, Gallstones, Dual pathology, Cholecystostomy

## Abstract

**Introduction:**

Acute abdominal pain accounts for 5% of all presentations to the emergency department (Stoker et al., 2009). Two of the most common causes are acute appendicitis and acute cholecystitis (Ferris et al., 2017).

**Presentation:**

A 70-year-old man presented with acute calculous cholecystitis. He subsequently deteriorated clinically and re-imaging revealed interval migration of stones from the biliary system to the appendix with resultant acute appendicitis.

**Discussion:**

Although both acute appendicitis and acute cholecystitis are common, dual pathology is rare. There are a small number of case reports of gallstones causing appendicitis (Vicari, 1964; Siegal et al., 1990; Meade, 1960).

**Conclusion:**

Our case report nicely illustrates.

a) The importance of considering dual pathology, especially when there is an unexpected change in the patient’s clinical status.

b) The CT features of two common acute surgical pathologies.

c) The value of cholecystostomy- performed in the Interventional Radiology suite- as a temporizing measure to allow the patient to recover from a critical illness.

## Highlights

• Case report of acute appendicitis and acute cholecystitis linked by migrating gallstones which illustrates.
• The importance of considering dual pathology.
• The CT features of two common causes of an acute abdomen.
• The value of cholecystostomy as a temporizing measure to allow the patient to recover from a critical illness.

## 1 Introduction

Acute abdominal pain accounts for 5% of all presentations to the emergency department [1]. Acute appendicitis and acute cholecystitis are two of the most common causes of the acute abdomen [2]. There are a small number of case reports of synchronous pathologies [6].

Imaging has a crucial role in management of these patients because clinical evaluation is often inaccurate. CT is the mainstay of acute imaging except for patients with suspected cholecystitis.

In line with SCARE [7] and PROCESS [8] guidelines, we present the case of a 70 year old male with acute calculous cholecystitis and stones in both the common bile duct and cystic duct. Subsequent clinical deterioration and re-imaging revealed interval migration of the stones to the appendix and resultant acute appendicitis. The patient was diagnosed and managed in our institution; a tertiary referral university teaching hospital.

## 2 Presentation of case

A 70-year-old male presented to our institution with a two-day history of right upper quadrant pain and fevers. On examination, he was found to have tenderness and guarding in the right upper and lower quadrants. He had a past medical history of abdominoperineal resection for colorectal cancer, hypertension and ischaemic heart disease.

**Fig. 1:**
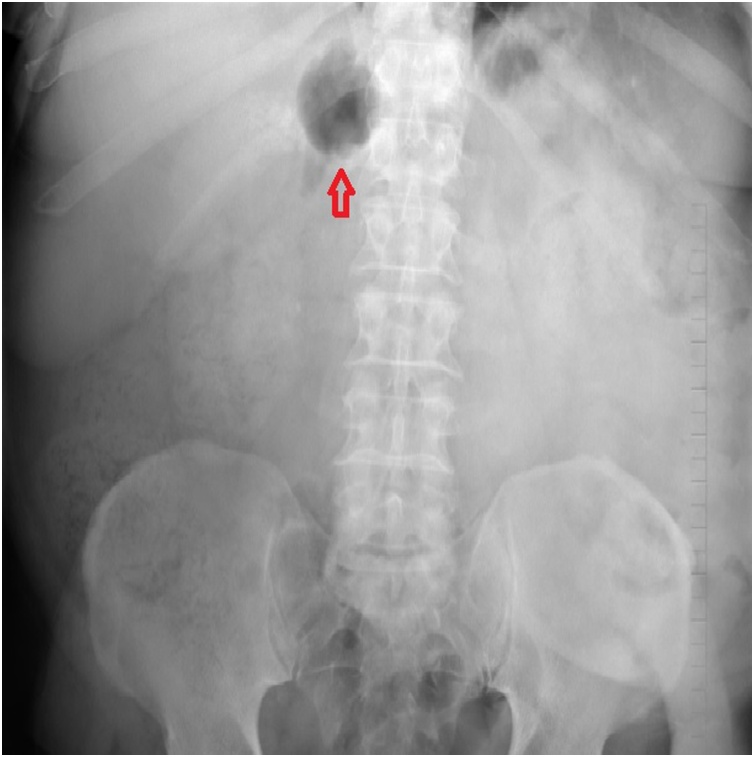
Abdominal x-ray showing a focally dilated bowel loop in the right upper quadrant (red arrow); the so called ‘sentinel loop’ sign.

The patient underwent an urgent abdominal x-ray followed by a CT abdomen/pelvis with intravenous contrast. The abdominal x-ray revealed a sentinel loop in the right upper quadrant (Fig. 1). This is a short segment of adynamic ileus adjacent to an acute inflammatory process.

**Fig. 2:**
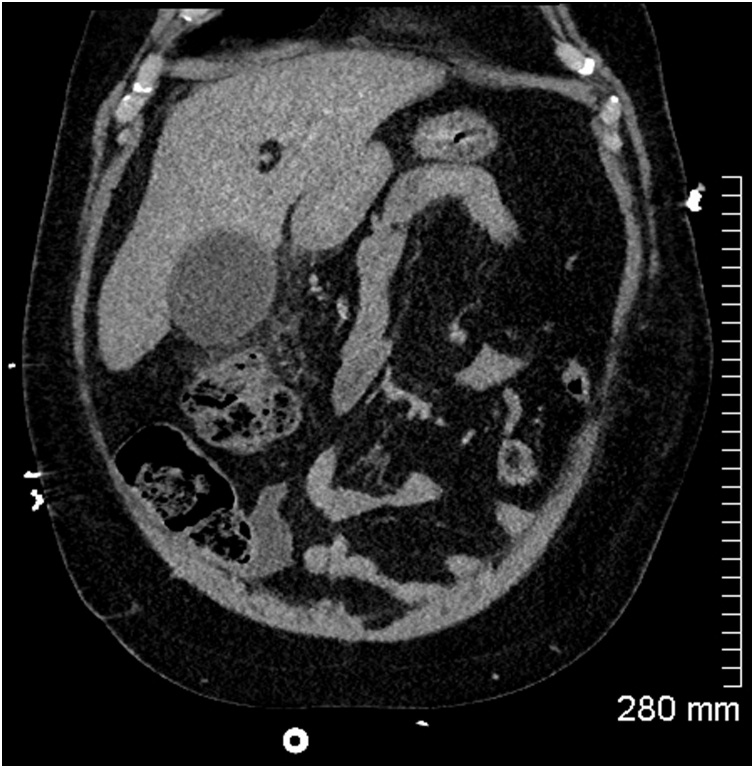
Coronal CT abdomen showing a distended gallbladder and pericholecystic fat stranding consistent with acute cholecystitis.

**Fig. 3:**
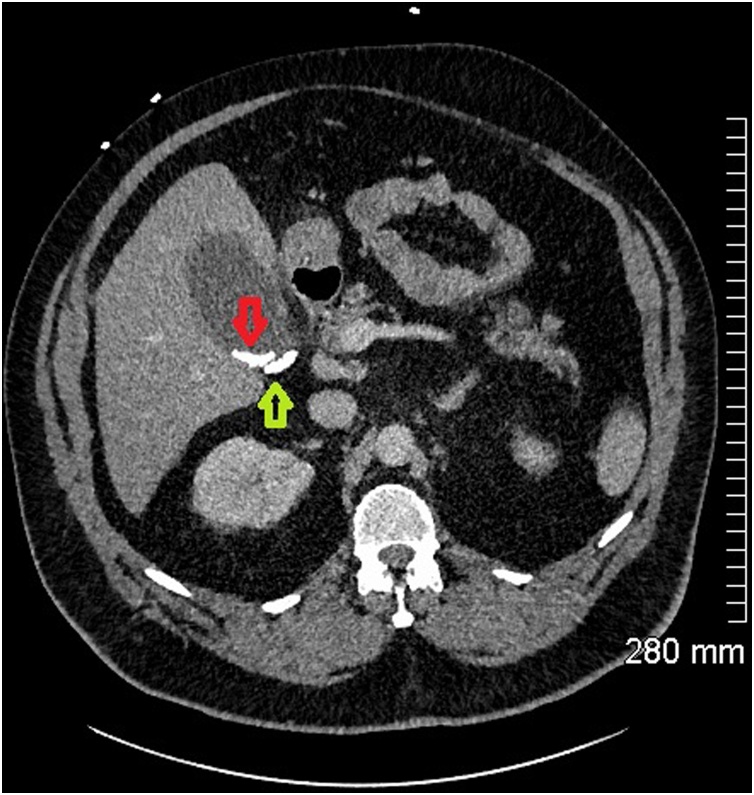
Axial CT abdomen showing calcified gallstones in the gallbladder (red arrow) and cystic duct (green arrow).

**Fig. 4:**
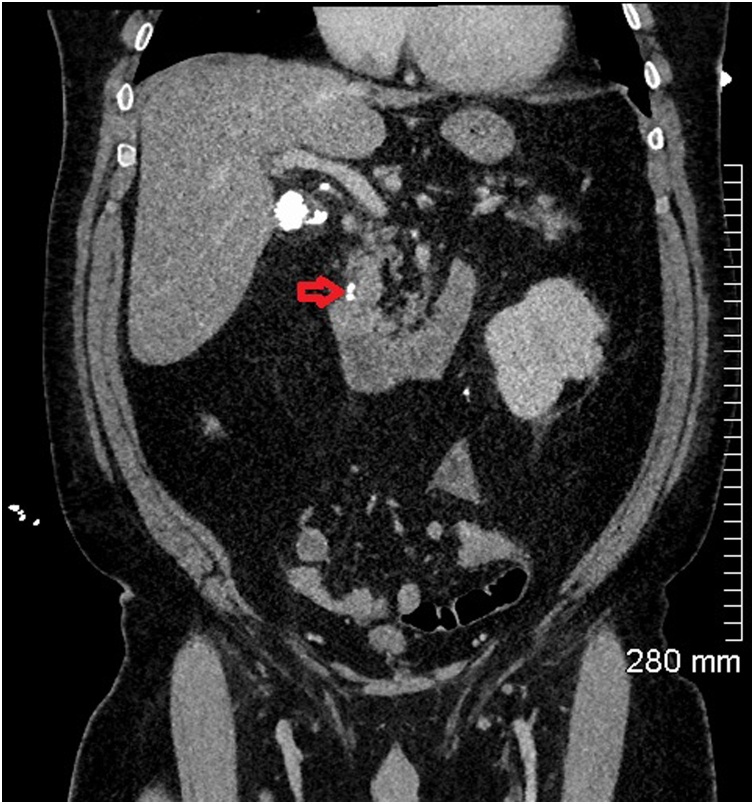
Coronal CT abdomen showing more calcified gallstones in the distal common bile duct (red arrow).

**Fig. 5:**
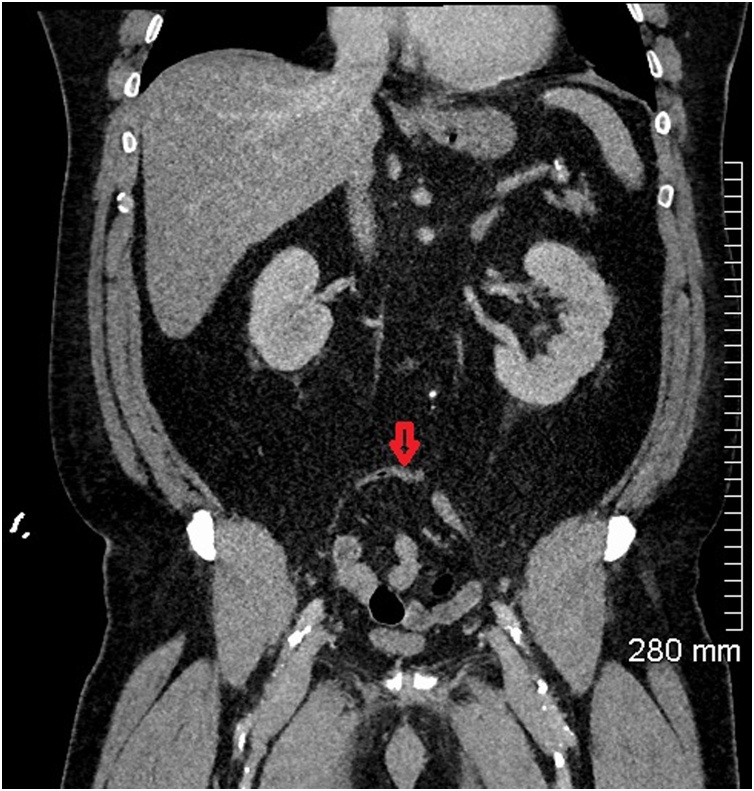
Coronal CT abdomen showing a normal appendix on admission (red arrow).

The CT revealed a distended gallbladder containing multiple calcified gallstones and extensive surrounding inflammatory fat stranding (Fig. 2). The appearance was consistent with acute cholecystitis. There were further stones in the cystic duct and common bile duct without any significant biliary dilation (Fig. 3, Fig. 4). The appendix was normal in appearance (Fig. 5).

**Fig. 6:**
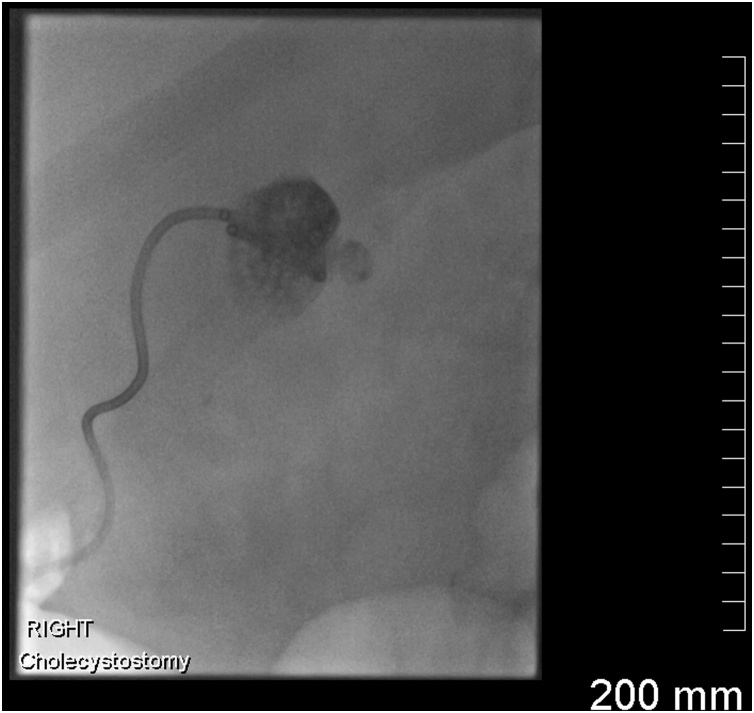
Fluoroscopic image showing a pigtail catheter in a satisfactory position in the gallbladder.

The patient underwent an ultrasound and fluoroscopy-guided cholecystostomy drain insertion in the Interventional Radiology suite (Fig. 6). The gallbladder was accessed via a transperitoneal approach without traversing any liver parenchyma because the patient had recently taken clopidogrel. An 8.5 French Mac-Loc drain was sutured in position and left on free drainage. A sample of bile was aspirated and sent for culture.

The patient was transferred to the Intensive Care Unit. He had a 21 day stay in the unit which was complicated by septic shock, ARDS, pulmonary oedema, atrial fibrillation and oliguric acute kidney injury requiring Continuous Veno-Venous Hemofiltration.

**Fig. 7:**
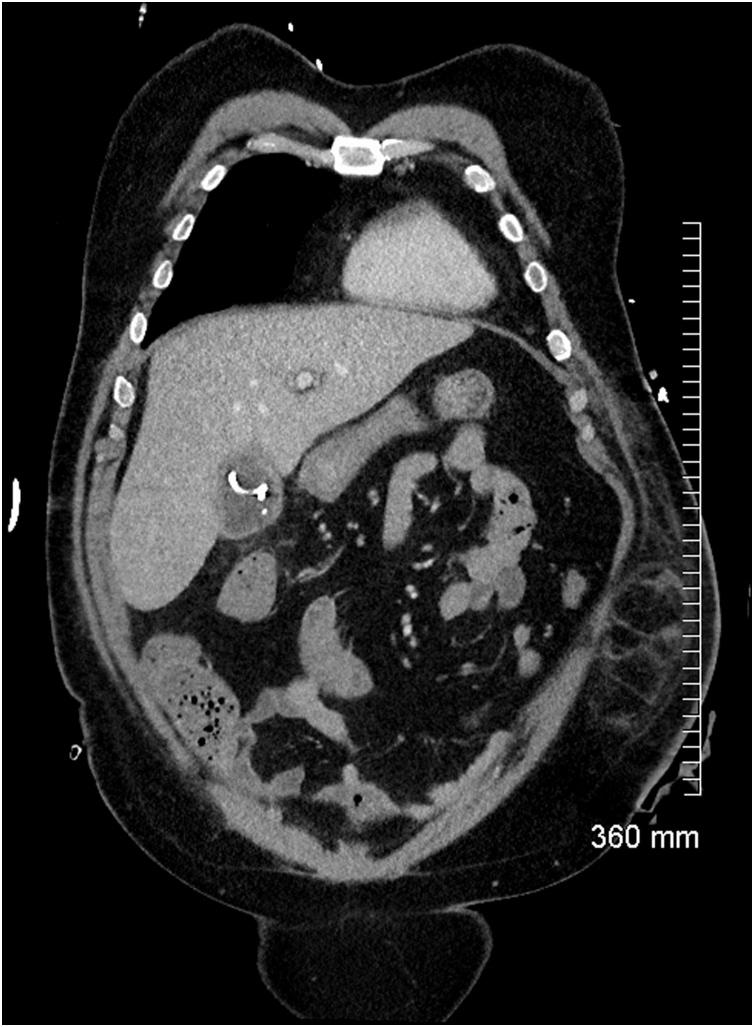
Coronal CT showing interval improvement in gallbladder distension and pericholecystic inflammatory changes with a cholecystostomy drain in situ.

**Fig. 8:**
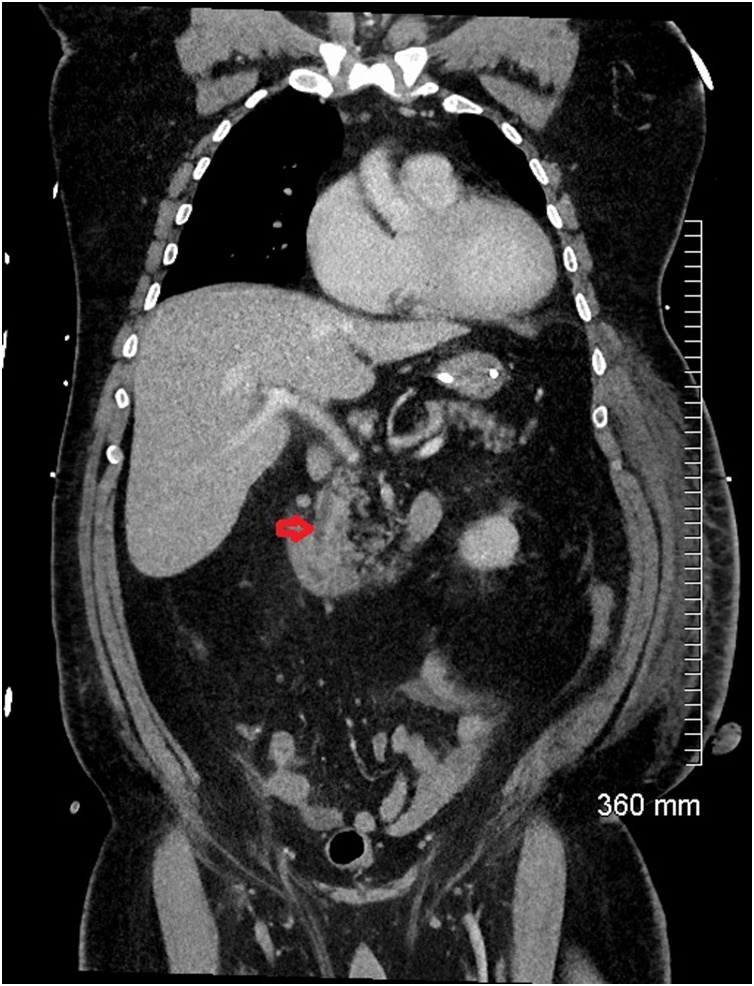
The stones in the cystic duct and common bile duct (red arrow) have migrated distally and the ducts are now clear.

**Fig. 9:**
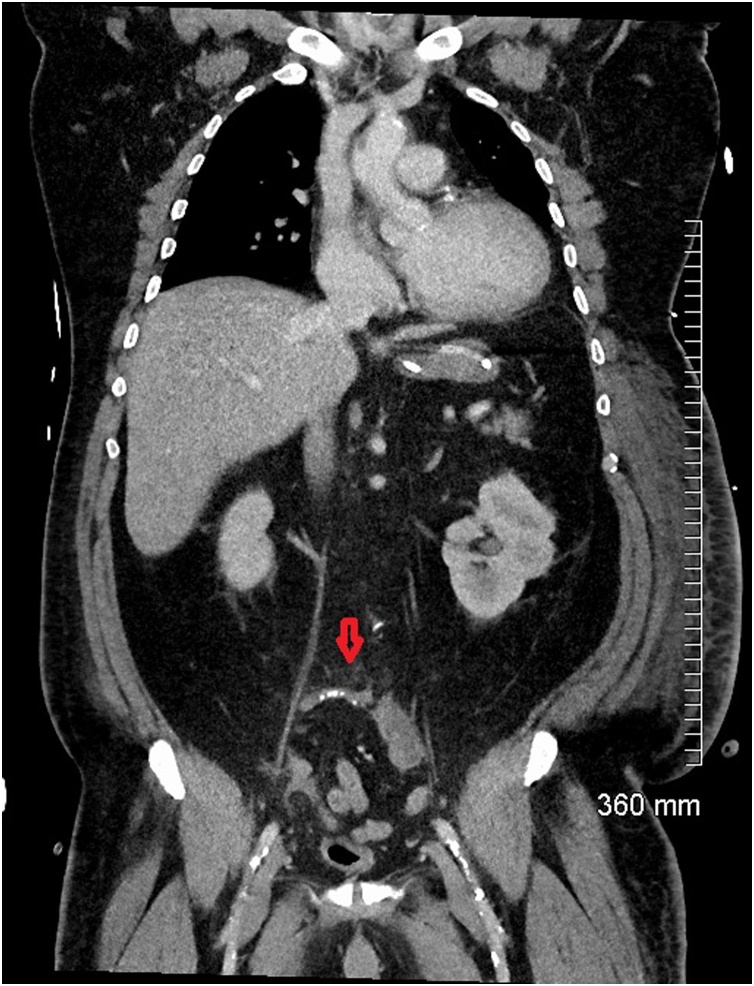
There are now multiple calcified densities within the appendix and there is new periappendiceal fat stranding (red arrow) consistent with acute appendicitis.

On day nine of his admission, his inotrope requirement increased and his abdomen became diffusely tender again. A repeat CT abdomen/pelvis was performed. It showed interval improvement in gallbladder distension and pericholecystic inflammatory change post cholecystostomy insertion (Fig. 7). However, there had been interval migration of the stones from the cystic and common bile duct to the appendix (Fig. 8). There was new periappendiceal fat stranding consistent with acute appendicitis (Fig. 9).

It was decided that the patient was not for surgery acutely due to his multiple other medical issues. He improved with conservative management and was transferred to the care of the Medicine for the Elderly team for a short period of rehabilitation prior to discharge. He was successfully discharged home two months after his admission. He will follow up with his surgical team to arrange an elective laparoscopic cholecystectomy as an outpatient.

## 3 Discussion

Acute appendicitis and acute cholecystitis are two of the most common causes of the acute abdomen [2]. A concurrent presentation is rare but the two pathologies do occasionally occur together [6] so dual pathology must be kept in mind.

It is hypothesized that the most likely pathogenesis is a primary appendicitis with bacterial translocation through the gut wall and seeding of the portal vein. Bacterial contamination of bile and impaired excretion then lead to acute cholecystitis [9]. This hypothesis is supported by the observation of hyperbilirubinemia in acute appendicitis [10]. Another study has shown an increased risk of clinically significant gallstones after appendicectomy [11].

Calcified deposits within the appendix are called appendicoliths. They contribute to the pathogenesis of acute appendicitis. The cause of their formation is uncertain but some case reports have implicated ingested foreign bodies or a dislodged gallstone eroding through the gallbladder [12].

There are a small number of case reports of gallstones causing appendicitis [[3], [4], [5]]. Cruz-Santiago et al. describe a case of gallstone ileus presenting as obstructive gangrenous appendicitis [13].

It is possible that CT in our patient has picked up an appendicitis that would have spontaneously resolved. Indeed, it has been suggested by some studies that the increasing use of sensitive imaging modalities such as CT has led to the increased detection of mild inflammation of the appendix which would have resolved without any medical or surgical intervention [[14], [15], [16]].

One study which evaluated a large group of patients which suspected appendicitis and followed up those who did not have surgery found a CT sensitivity of 98.5%, a specificity of 98%, a positive predictive value of 93.9% and a negative predictive value of 99.5% for the diagnosis [17].

Our case represents an unusual example of stones migrating from the common bile duct to the appendix and resultant dual pathology. His biliary sepsis was successfully managed with cholecystostomy drain insertion and intravenous antibiotics. The patient was discharged home with a view to elective cholecystectomy as an outpatient.

## 4 Conclusion

Our case illustrates a nice example of both acute appendicitis and acute cholecystitis- two of the most common causes of an acute abdomen- linked by migrating gallstones. The case reminds us to consider dual pathology, especially when there is an unexpected change in the patient’s clinical status. The case illustrates the CT features of both pathologies as well as the sentinel loop sign on abdominal x-ray. The case also demonstrates the value of cholecystostomy-performed in the Interventional Radiology suite- as a temporizing measure to allow the patient to recover from a critical illness.

## Declaration of Competing Interest

The authors report no declarations of interest.

## Funding

Not applicable.

## Ethical approval

Not applicable.

## Consent

Written informed consent was obtained from the patient for publication of this case report and accompanying images. A copy of the written consent is available for review by the Editor-in-Chief of this journal on request.

## Author contribution

Gerard Lambe conceived the idea for the case and drafted the manuscript.

Mark Murphy, Hazel O’ Neill and Simon Doran assisted with preparation and proofreading of the figures and the final manuscript.

Noel E Donlon provided clinical input from the Department of Surgery.

Niall McEniff provided specialist radiology input and comments.

## Registration of research studies

Not applicable.

## Guarantor

Gerard Lambe.

## Provenance and peer review

Not commissioned, externally peer reviewed.
